# Identification and Management of Abdominal Aortic Dissection With Concurrent Aneurysm

**DOI:** 10.7759/cureus.16621

**Published:** 2021-07-25

**Authors:** Adel Hanandeh, Mitchell Weaver, Fadi Baidoun

**Affiliations:** 1 General Surgery, Henry Ford Health System, Detroit, USA; 2 Vascular Surgery, Henry Ford Health System, Detroit, USA

**Keywords:** isolated abdominal aortic dissection, aortic dissection, abdominal aortic aneurysms, management of abdominal aortic aneurysms, abdominal aortic dissection with concurrent aneurysm

## Abstract

The incidence of acute aortic dissection ranges from 2.6 to 3.5 per 100,000 people per year. An abdominal aortic dissection is known to be the rarest of all types of aortic dissection, with high morbidity and mortality rates. In this case report, we are hoping to shed light on this unusual entity, its etiology, and management options.

## Introduction

Abdominal aortic dissection is a rare pathology of the abdominal aorta, accounting for only 1-4% of aortic pathologies. There are multiple etiologies described in the literature for this rare condition including spontaneous, traumatic, and iatrogenic, with first being the most common [1]. Concurrent aortic dissection along with aneurysm has been described in less than 40% of the cases [2]. In this case report, we are describing a spontaneous case of abdominal aortic dissection with a concomitant aneurysm in a healthy patient with no known comorbidities that was treated with an open retroperitoneal abdominal aortic repair.

## Case presentation

A 65-year-old female with no past medical history presented to the emergency department on 5/12/2021 with a complaint of periumbilical abdominal pain that radiated to the back. The pain was described to be tearing and sharp in nature, measuring 10/10 on the pain scale. The pain started suddenly after waking up earlier that morning. The patient presented six hours from the onset of symptoms. Upon presentation, the patient was evaluated by the emergency room staff vitals were obtained, and appeared to be within normal limits except for blood pressure in 150's systolic and over 90's diastolic. A full set of labs were obtained, which indicated a white blood cell count of 8.1 K/uL, hemoglobin of 15.5 g/dl, hematocrit of 45.4%, and platelets of 198 K/uL. Basic metabolic panel and coagulation studies were within the normal range. 

**Figure 1:**
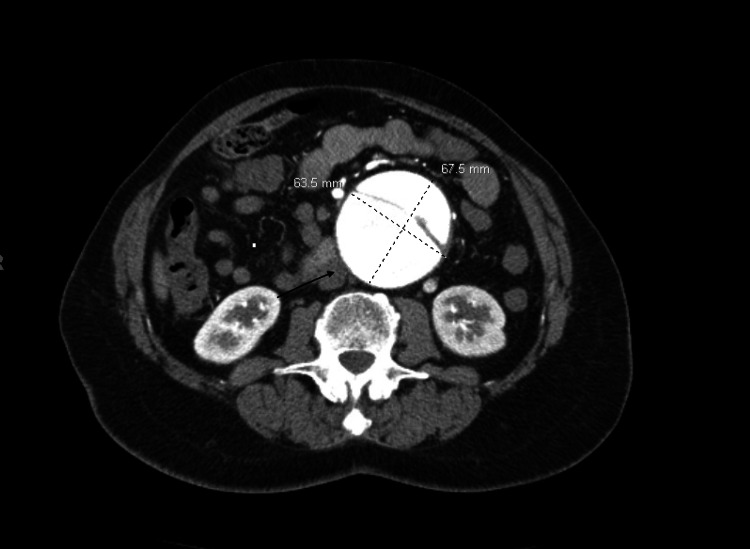
Computed Tomography of the Abdomen Illustrating the Abdominal Aortic Dissection and Aneurysm

**Figure 2:**
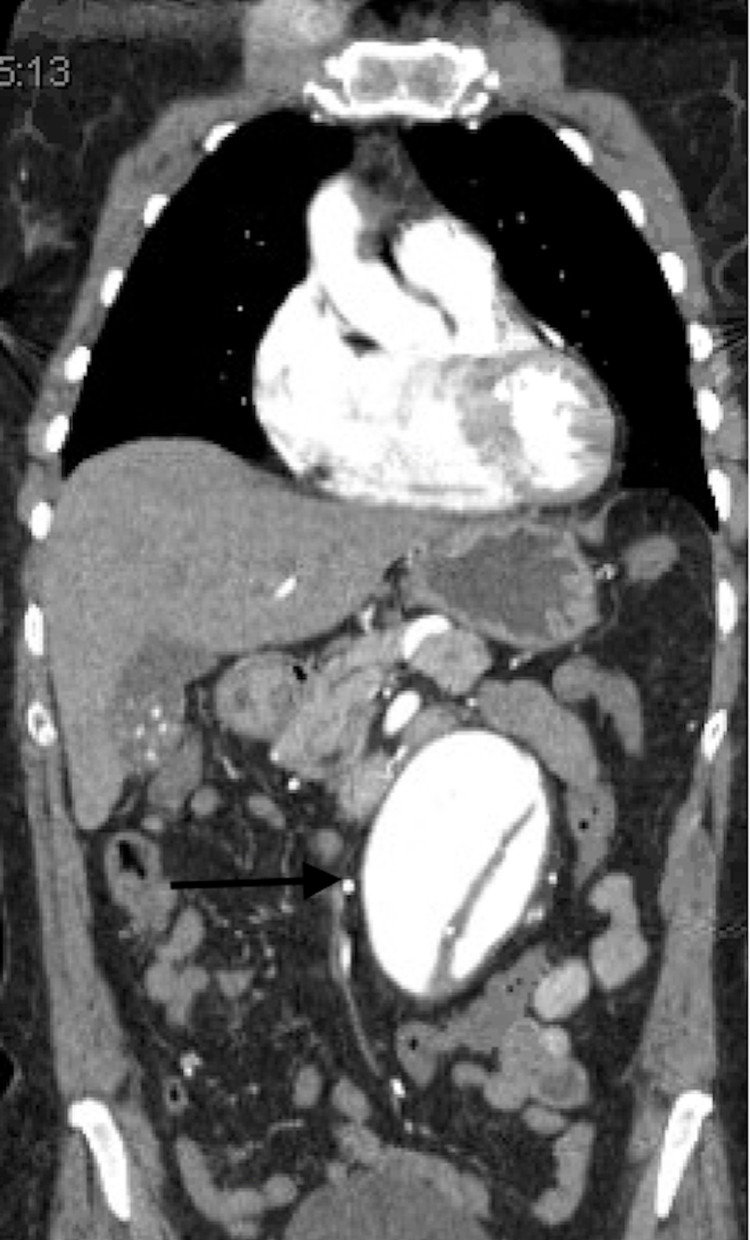
Computed Tomography of the Abdomen Illustrating the Abdominal Aortic Dissection and Aneurysm

**Figure 3:**
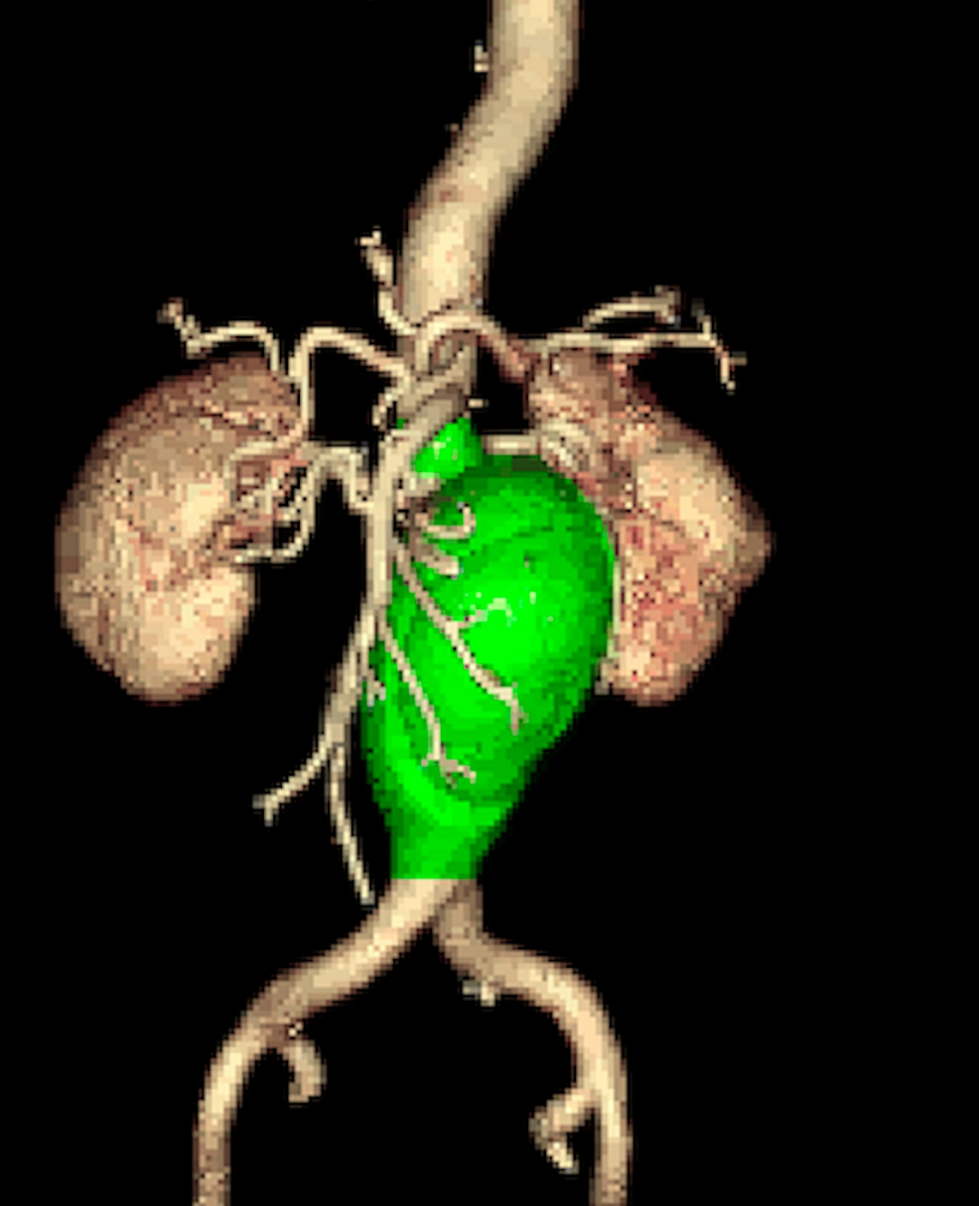
Three-Dimensional Image of the Abdominal Aortic Aneurysm

Computed tomography chest, abdomen, and a pelvis with intravenous contrast were obtained and indicated infrarenal abdominal aortic tortuosity as well as 68 mm abdominal aortic aneurysm with dissection. The infrarenal abdominal aorta was proximally tortuous leading into the fusiform infrarenal abdominal aorta aneurysm and short segment dissection with a wide intimal defect with equal opacification of the true and false lumen. The aneurysm extended to the level of the bifurcation. There were two left and one right renal arteries that were patent. Vessels were coursing along the left lateral aspect of the aneurysm (Figure 1-3). There were concerns for impending rupture.

**Figure 4:**
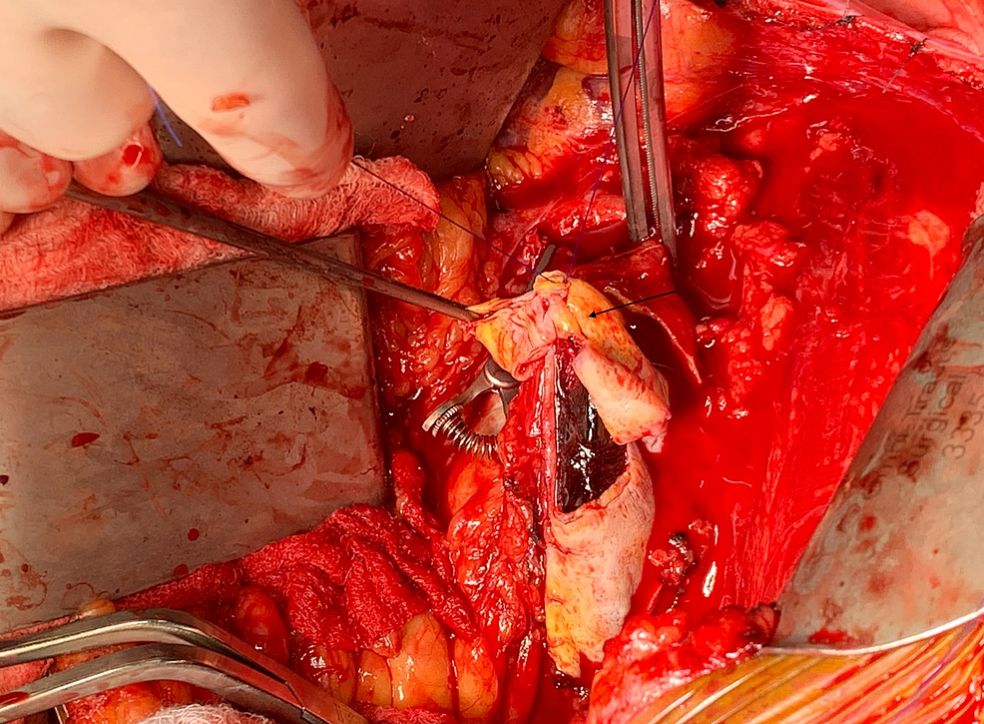
Intra-operative Image of the Abdominal Aortic Intimal Flap

Given the concerns for impending rupture, the patient required immediate surgical intervention. Unfortunately, the patient was not a candidate for endovascular therapy due to the juxta-renal nature of the aneurysm and the lack of adequate proximal neck. Therefore, the patient underwent an open retroperitoneal repair, where she was found to have a large dissection with a prominent intimal flap as indicated in Figure 4. A 16 mm Dacron graft was placed and the aneurysm sac was closed over the graft using interrupted 3-0 vicryl sutures. The patient tolerated the procedure well, was successfully extubated, and was transferred to the ICU for hemodynamic monitoring. The patient did very well and was successfully discharged on post-operative day three. 

## Discussion

Spontaneous isolated abdominal aortic dissection (IAAD) is a rare entity, most common among male patients with an average age of 67.7 ± 13.3 years old [2]. In patients with IAAD, the most common comorbidities are hypertension and pre-existing aortic aneurysm which can be found in up to 40% of patients. The most common location of such dissection has been reported in the literature to be at the level of the renal arteries and infrarenal. While supra-renal dissection nidus being the rarest of all [3].

The pathophysiology of aortic dissection entails several variants including aortic intramural hematoma, intimal tear without hematoma, and penetrating atherosclerotic ulcer. Aortic intramural hematoma is characterized by blood in the wall of the aorta in the absence of an intimal tear that accounts for 5-13%. The false channel is likely produced by a rupture of the vasa vasorum into the media of the aortic wall and can occur in the absence of significant atherosclerosis. 

Intimal tear without hematoma is an uncommon variant of aortic dissection that is characterized by an intimal tear associated with exposure of the underlying aortic media or adventitial layers, most commonly associated with blunt aortic injury with a focal tear. Finally, penetrating atherosclerotic ulcers are often associated with aortic intramural hematoma and can also lead to aortic dissection or perforation [4].

There are multiple management options for aortic abdominal dissection and aneurysms that have been proposed. Including open, endovascular and conservative approaches depending on the size of the dissection, concurrent aneurysm size, and the location of the dissection in relation to the renal arteries [1-3].

In a meta-analysis study by Jonker and his colleagues which included 92 patients with abdominal dissection, in which they concluded that concurrent aortic aneurysms were more often associated with spontaneous dissections (p =0.002). Aortic rupture occurred in 10 percent of abdominal aortic dissection (AADs). Meanwhile, management included open repair (50%), endovascular repair (21%), and conservative medical treatment (29%). With in-hospital mortality of 4% overall, 2% in the open repair group, 0% in endovascular, and 8% with conservative treatment. They concluded that endovascular therapy was associated with a low risk of mortality and major complications compared to open repair or conservative treatment [3]. 

In another systematic review of isolated abdominal aortic, a meta-analysis of 17 studies, single-arm-based that was performed by Wu et al. [5] reported that the prevalence of isolated abdominal aortic dissection (IAAD) among cases of aortic dissection overall, type B aortic dissection, and type A aortic dissection was 1.7% [95% confidence interval (CI), 0.9%-3.4%], 4.1% (95% CI, 2.5%-6.6%), and 2.0% (95% CI, 0.7%-3.9%), respectively. Abdominal pain was the most common symptom (50.8%), followed by back pain (30.5%), and chest pain (21.7%). Up to 41% of the patients with IAAD did not present with any clinical symptoms, and up to 71% of these patients had negative findings on physical examination. They also concluded that three of the most common risk factors for IAAD were hypertension, hyperlipidemia, and smoking. Most cases of IAAD were limited to the aorta inferior to the renal arteries (81.7%), with an average aortic diameter of 4 cm [5]. They concluded that there was no statistically significant difference between open surgery, endovascular aortic repair, and conservative management for both early and late mortalities.

Finally, the Investigation of Stent-grafts in Aortic Dissection with extended length of follow-up (INSTEAD XL) trial on uncomplicated type B thoracic aortic dissections indicated that in patients with IAAD who are hemodynamically stable with little clinical signs and complications, there is a role for conservative medical management with careful surveillance. They also concluded that endovascular management in addition to optimal medical treatment is associated with improved five-year aorta-specific survival and the delayed disease progression. In stable abdominal dissection with suitable anatomy, preemptive endovascular therapy should be considered to improve late outcomes [6].

## Conclusions

Abdominal aortic dissection is a very rare pathology, associated aneurysms are very common and increase the risk for impending rupture, therefore we recommend immediate surgical treatment via an endovascular or open approach based on the anatomic location and feasibility.
